# Intra- and inter-rater reliability of an electronic health record audit used in a chiropractic teaching clinic system: an observational study

**DOI:** 10.1186/s12913-021-06745-1

**Published:** 2021-07-28

**Authors:** H. Stephen Injeyan, Sheilah Hogg-Johnson, Sean Abdulla, Ngai Chow, Jocelyn Cox, Anthony Ridding, Craig Jacobs

**Affiliations:** 1 Research and Clinical Education, Canadian Memorial Chiropractic College, 6100 Leslie Street, Toronto, Ontario M2H 3J1 Canada; 2 Graduate Education and Research Programs, Canadian Memorial Chiropractic College, Toronto, Ontario Canada; 3 Division of Clinical Education, Canadian Memorial Chiropractic College, Toronto, Ontario Canada

**Keywords:** File audit, Inter-rater, Reliability, Chiropractic, Electronic health record EHR, Standards

## Abstract

**Background:**

There is a dearth of information about health education clinical file audits in the context of completeness of records and demonstrating program-wide competency achievement. We report on the reliability of an audit instrument used for electronic health record (EHR) audits in the clinics of a chiropractic college in Canada.

**Methods:**

The instrument is a checklist built within an electronic software application designed to pull data automatically from the EHR. It consists of a combination of 61 objective (*n* = 20) and subjective (*n* = 41) elements, representing domains of standards of practice, accreditation and in-house educational standards. Trained auditors provide responses to the elements and the software yields scores indicating the quality of clinical record per file.

A convenience sample of 24 files, drawn randomly from the roster of 22 clinicians, were divided into three groups of eight to be completed by one of three auditors in the span of 1 week, at the end of which they were transferred to another auditor. There were four audit cycles; audits from cycles 1 and 4 were used to assess intra-rater (test-retest) reliability and audits from cycles 1, 2 and 3 were used to assess inter-rater reliability. Percent agreement (PA) and Kappa statistics (K) were used as outcomes. Scatter plots and intraclass correlation (ICC) coefficients were used to assess standards of practice, accreditation, and overall audit scores.

**Results:**

Across all 3 auditors test-retest reliability for objective items was PA 89% and K 0.75, and for subjective items PA 82% and K 0.63. In contrast, inter-rater reliability was moderate at PA 82% and K 0.59, and PA 70% and K 0.44 for objective and subjective items, respectively. Element analysis indicated a wide range of PA and K values inter-rater reliability of many elements being rated as poor. ICC coefficient calculations indicated moderate reliability for the domains of standards of practice, accreditation, and overall file scores.

**Conclusion:**

The file audit process has substantial test-retest reliability and moderate inter-rater reliability. Recommendations are made to improve reliability outcomes. These include modifying the audit checklist with a view of improving clarity of elements, and enhancing uniformity of auditor responses by increased training aided by preparation of an audit guidebook.

**Supplementary Information:**

The online version contains supplementary material available at 10.1186/s12913-021-06745-1.

## Introduction

Quality assurance (QA), through regulatory and accreditation activities, and the regular review of clinical records with a view of providing audit-feedback to stakeholders has been recognized as an important contributor to improving clinicians’ performance and ultimately patient care [1, 2]. The reliability of the audit instrument in enhancing validity and overall effectiveness of audit-feedback is essential [1, 3–5].

Efforts have been devoted to the study of reliability of audits of the clinical record through chart abstraction, particularly in the medical field [6]. The common approach has been scrutiny of the record using specially developed abstraction tools to examine specific aspects of the health record with a view of generating data for scrutinizing patient outcomes or assessing effectiveness of intervention programs. Many such examples are available from different medical disciplines [4, 7–11], as well as allied health care professions [12–16]. While reliability of audit tools has been specified in most cases, some audit studies have utilized survey type instruments and/or interviews [14, 15] with no indication of reliability data.

There are several examples of file audit studies in the chiropractic profession both in the context of professional practice [8, 17, 18] or in teaching institutions [19, 20]; however, reliability assessments of audit instruments and/or processes used are lacking. In Ontario, Canada, the chiropractic regulator performs file (chart) audits of practicing chiropractors in the context of their “Peer and Practice Assessment Program” [21]. The results of such audits are communicated to individual practitioners; however, our search of the peer reviewed literature revealed no published literature about the methods used, and the outcomes of such audits. A recent RAND investigation [22] used a file abstraction method to research the appropriateness of care in the chiropractic setting in the USA. The findings suggested a need for improved detail and rationale for care. Similar studies in the chiropractic educational setting are lacking. Furthermore, the extent to which legislative and/or accrediting standards are incorporated into educational programs, in health care generally, and their impact on student trainees is not known [8].

Thus, to our knowledge, there is a great deal of literature available on chart review and abstraction with a view of extracting data as health outcomes indicators or to provide the basis for research in specific areas. However, there is a dearth of information about audits of health education outcomes in the context of completeness of records and demonstrating program-wide competency achievement.

During recent decades, the use of the electronic health record (EHR) for the documentation of the clinical record has become commonplace [23]. With this, there is the expectation that clinical file reviews, for the purpose of quality improvement, would become easier. Although pitfalls in the use of the EHR have been identified and recognized widely [24, 25] nevertheless the advantages of the EHR in helping to standardize the health record and streamline the process of auditing the clinical record has been noted [26]. However, the EHR is complex, and it may require navigation to locate specific data. In the absence of a uniform approach by auditors to locate information, assessment of inter-rater reliability may be subject to error [27].

Since the early 2000s, the use of the EHR in chiropractic practices has become common [28], and most chiropractic colleges in North America have transitioned to EHR [29]. The outpatient clinics of the Canadian Memorial Chiropractic College (CMCC) transitioned to an EHR system in 2013 utilizing a specifically modified software currently named Indivicare Production [30]. Subsequently, audits of the EHR have been performed regularly using an in-house developed audit instrument specific for this purpose. However, to date the reliability of the instrument used to perform the audits has not been assessed. In this manuscript, we briefly describe the process of developing the audit instrument used for audits in our clinics and determine its intra- (test-retest) and inter-rater reliability. We believe this will contribute to the effectiveness of the audit process, helping to provide valid feedback to clinicians and contribute to curricular development.

## Methods

### The audit instrument

An electronic software application, developed by CMCC’s IT specialists, is used to pull data automatically from the EHR system used at CMCC clinics. It was designed to facilitate the file audit process and increase efficiency by eliminating the need for data extractors, including file auditors, to toggle between two systems; the EHR, and a data collecting system. A checklist, henceforth referred to as the “audit instrument”, is built within the software. The audit instrument was developed through discussions and consultations involving clinical administrators and information technology specialists. Furthermore, several sessions were organised to assess and ensure the face and content validity of the instrument. These brought together the clinic Dean, two clinic Directors, and potential auditors with clinical experience familiar with the EHR used in CMCC’s clinics. The salient aspects of the instrument such as elements included, and their categorization were discussed, and agreed upon by consensus. A draft version of the instrument was then used to pilot the process by several potential auditors and clinicians. Following further discussions and “fine-tuning”, the completed version of the instrument had 10 sections (history, physical exam, diagnosis, plan of management, goals, prognosis, report of findings and consent, case report, documentation, re-evaluation) comprising 61 elements, and covered the standards of practice required by the regulatory body (College of Chiropractors of Ontario [31]) as well as Accreditation Educational standards stipulated by the Federation of Canadian Chiropractic [32]. In addition, several elements were included as educational items consistent with the institution’s curricular competencies which were not captured in the other standards. Twenty elements are characterized as being objective and are scored as “Yes”/“No” or “not applicable”, while the remaining 41 elements are subjective in nature and are scored as “complete,” “partially complete,” “absent,” or “not applicable” (Additional file 1). The estimated average time to audit a file was 30 min.

### Scoring

Within the instrument, elements are specifically designated as being required “standard of practice” or “accreditation standard” elements, with the majority of elements belonging to both groups. Responses to elements are routinely combined to create three scores within the audit software application. The Overall Score combines all responses by summing them and converting to a percentage. Yes/no items contribute 2 for yes and 0 for no, and complete/partial/absent items contribute 2 for complete, 1 for partial and 0 for absent. Not applicable elements are removed from the calculation. A Standards of Practice score is derived by summing and converting to a percentage the 38 items reflecting standards of practice. An Accreditation score is derived by summing and converting to a percentage the 37 items related to accreditation standards.

### Selection of files for audit

As a matter of routine, CMCC clinic management performs audits on two rotating cycles per year. At each cycle six files are drawn randomly from the roster of each of the 22 clinicians utilizing the EHR yielding a total sample of 132 patient files available for audit.

For this study, of the available132 files, a convenience sample of 24 (18%) files was drawn randomly using a randomization algorithm built in the software.

Three auditors, who were trained for the use of the instrument, and had been doing file audits of the EHR regularly, consented to participate in the study. They were all licenced chiropractors in practice for an average of 5 years as well as being on CMCC faculty. The 24 files were divided into three groups of eight. There were four cycles of auditing, of one-week duration each. At the end of each week, the administrator of the study (CJ) reallocated each group of files to a different auditor. This was repeated until all 4 cycles were completed among the three auditors. Each auditor reviewed the same set of files in cycles 1 and 4 for test-retest reliability. The audits from cycles 1, 2 and 3 were used for inter-rater reliability. A timing feature of the audit software captured the length of time, in minutes, that an auditor took to complete the audit. These data were extracted after study completion for all 96 audits (24 files × 4 audits per file).

Measures were taken to mitigate the risk of compromising confidentiality of stakeholders including patients, clinicians, interns, and auditors. While patient, clinician, and intern names could not be concealed from the auditors or the audit administrator (CJ), as they are part of the clinical record, researchers involved in data analysis were blinded. They were also blinded to the identity of the auditors. Raw audit data were anonymized by the IT collaborator and transferred to the research team for analysis. Thus 24 files included in the analysis were identifiable by file numbers only. Auditor names were concealed by the study administrator by assigning each auditor the number 1, 2, or 3.

The results of one auditor could not be seen by another, thus eliminating observer bias. Potential communication amongst the three auditors was mitigated by providing strict instructions, at the outset, to avoid any discussion of the files during the audit period. All anonymized data were stored electronically and were password protected.

### Data analysis

#### Test-retest reliability

There were 24 files (8 files per rater) with 61 elements (items) yielding 1464 items overall assessed twice for test-retest reliability, with 480 of these from yes/no/not applicable items and 984 related to complete/partial/absent/not applicable items. A three-by-three cross-tabulation of the 20 yes/no/not applicable items was constructed pooling across all auditors and items and then separate tables were constructed for the three auditors. Similarly, a four-by-four cross-tabulation of the 41 complete/partial/absent/not applicable items was constructed pooling across all auditors and items, and then separate tables were constructed for each of the auditors. For each of these tables, the percentage agreement (PA) on test and retest and the kappa (K) statistic [33] with 95% confidence intervals (CI) were calculated. Percent agreement (PA) and kappa (K) are commonly used to measure test-retest and inter-rater reliability with categorical measures. K is a measure of agreement corrected for chance, with a potential scale of values from 0.00–1.00 with higher values indicating better agreement. A threshold of K 0.6 has been suggested for “substantial” level of intra- and inter-rater reliability [34].

Similar tables were constructed for each individual item with % agreement and kappa statistics calculated based on the 24 observations in the cross-tabulations (one for each file in the study).

For each patient file, for each audit, the items were combined into three scores pertaining to the domains of standards of practice, accreditation standards as well as overall file scores as detailed above. Scores were constructed by assigning values of 0 and 2 to no and yes responses, respectively, and values of 0, 1 and 2 to absent, partial and complete responses, respectively and averaging these across all items that pertained to a domain. Items judged not applicable were not included in the scores. Scatter plots of the three constructed scores for the 24 files from test and retest were made to observe the level of agreement, and intraclass correlation coefficients (ICC) (2,1), with 95% CI were derived [35].

#### Inter-rater reliability

Audit responses from cycles 1, 2 and 3 were used to assess inter-rater reliability. The process was similar to that used for test-retest reliability except there are three ratings for each item for each file. To examine agreement across three raters we looked at each pair combination separately (e.g., Auditor 1 versus Auditor 2, Auditor 1 versus Auditor 3 and Auditor 2 versus Auditor 3) with agreement statistics for the pair, and then the three-rater kappa with 95% CI or three rater ICC with 95% CI was also computed to look at overall agreement.

#### Audit times

The time taken to complete the audits was described using mean, standard deviation, minimum and maximum, and audit times between auditors and between files were compared using Analysis of Variance.

#### Statistical software

Statistical analysis was generated using SAS software v9.4. (Copyright© 2012–2018, SAS Institute Inc., Cary, NC, USA). The graphical analysis and intraclass correlation coefficients were generated using R [36] and the R package “psych” [37].

The institutional Research Ethics Board of the Canadian Memorial Chiropractic College approved the study (#1904B04).

## Results

### Auditor perspectives

All three auditors completed their respective reviews within the designated cycle. Electronic data extracted by the IT administrator was anonymized and submitted for analysis. For each of the three auditors, test-retest reliability with respect to both 20 objective, and 41 subjective items is shown in Table 1.

**Table 1:** Summary of Intra-auditor (test-retest) reliability

Auditor	Objective items		Subjective items	
	% agreement (95%CI)	Kappa (95%CI)	% agreement (95%CI)	Kappa (95%CI)
1	88 (83, 93)	0.80 (0.72, 0.88)	86 (82, 89)	0.76 (0.70, 0.82)
2	93 (88, 97)	0.71 (0.56, 0.87)	84 (80, 88)	0.65 (0.57, 0.74)
3	86 (80, 91)	0.63 (0.50, 0.76)	75 (71, 80)	0.49 (0.41, 0.59)
Overall	89 (86, 92)	0.75 (0.69, 0.81)	82 (79, 84)	0.63 (0.61, 0.70)

Overall, combining data from all three auditors, yielded 89% agreement with K 0.75 (95% CI 0.60, 0.81) for the objective items, and 82% agreement with K 0.63 (95% CI 0.57, 0.74) for subjective items (Table 1).

Analysis of the data for inter-rater reliability yielded overall agreement of 82 and 70% with K values of 0.59 (95% CI 0.53, 0.66) and 0.44 (95% CI 0.40, 0.48) for objective and subjective elements, respectively (Table 2).

**Table 2:** Summary of Inter-auditor reliability

Auditors	Objective items (n = 20)		Subjective items (*n* = 41)	
	% agreement (95%CI)	Kappa (95%CI)	% agreement (95%CI)	Kappa (95%CI)
1 and 2	88 (85, 91)	0.72 (0.65, 0.78)	71 (68, 73)	0.46 (0.41, 0.51)
1 and 3	78 (74, 81)	0.50 (0.43, 0.57)	73 (70, 75)	0.47 (0.42, 0.52)
2 and 3	81 (77, 85)	0.57 (0.50, 0.64)	67 (64, 69)	0.41 (0.36, 0.45)
Three-auditor	82 (80, 84)	0.59 (0.53, 0.66)	70 (69, 72)	0.44 (0.40, 0.48)

### Element analysis

Test-retest reliability was examined with respect to each specific element encompassing all 24 files (Table 3). Overall, 52 of 61 items (85%) had a minimum test-retest agreement of 70%. Of these, 19/20 (95%) in the objective category had an agreement level of 70% or better, compared to 33/41 (80%) items in the subjective category. For the Kappa statistics, 32/61 (52%) achieved an overall K value of 0.6 or higher (range = − 0.06 to 1.00) (Table 3).

**Table 3:** Item by Item Test-Retest & Inter-rater Reliability Showing % Agreement and Kappa Statistics (*N* = 24 Files)

Section	Element	Response type	SP	AS	ES	Test-re-test		Inter-rater	
						% agree	kappa	% agree	kappa
History	Demographic Data Complete	subjective	S			87.50	0.3739	84.72	0.1410
History	Privacy Form present and complete	subjective			E	83.33	0.7467	59.72	0.3737
History	New Patient Form present and complete	subjective	S			66.67	0.3725	48.61	0.0111
History	Health Status Survey/Pain Diagram reviewed by clinician	objective	S			75.00	0.4000	66.67	0.2334
History	Outcome Measures appropriate	objective	S	A		79.17	0.5699	72.22	0.4198
History	Past Health History	subjective	S	A		62.50	−0.0385	66.67	0.1111
History	Family Health History	subjective	S	A		62.50	0.4114	47.22	0.1582
History	Social History	subjective	S	A		87.50	0.6066	66.67	0.0759
History	Flags	subjective		A		54.17	0.2000	37.50	−0.0679
History	Primary Complaint Complete	subjective	S	A		83.33	0.5556	50.00	−0.2101
History	Secondary Complaint (if applicable)	subjective	S			62.50	0.3684	45.83	0.1063
History	Consent to Physical Examination obtained and documented	subjective	S	A		100.00	1.0000	97.22	0.9434
History	Differential Diagnoses rendered	subjective	S	A		83.34	0.6129	75.00	0.4311
History	Complexity Completed	objective		A		87.50	0.6538	75.00	0.3069
Physical Examination	Observation/posture	subjective	S			95.84	0.8710	90.28	0.7483
Physical Examination	Vitals	subjective	S	A		75.00	0.4947	58.33	0.3411
Physical Examination	Range of Motion	subjective			E	79.17	0.5219	68.06	0.3196
Physical Examination	Palpation	subjective			E	100.00	1.0000	100.00	1.0000
Physical Examination	Orthopaedic Procedures	subjective	S	A		95.84	0.7857	88.89	0.5590
Physical Examination	Correlates with history	subjective		A		95.84	0.7857	91.67	0.6364
Physical Examination	Neurologic Procedures	subjective	S	A		95.84	0.8909	94.44	0.8633
Physical Examination	Procedures sufficient to: Exclude differential diagnoses	subjective		A		87.51	0.5714	86.11	0.4865
Physical Examination	Procedures sufficient to: Render clinical diagnosis	subjective	S	A		100.00	1.0000	91.67	0.5917
Physical Examination	Complexity Completed	objective		A		91.67	0.7612	86.11	0.5740
Diagnosis	Diagnoses rendered	subjective	S	A		95.84	0.8182	97.22	0.8786
Diagnosis	Appropriate and supported by findings	subjective		A		95.84	0.7857	80.56	0.3429
Diagnosis	Complexity Completed	objective		A		95.84	0.8806	91.67	0.7690
Plan of Management	Further Evaluation	subjective	S			87.51	0.5294	80.56	0.4661
Plan of Management	Frequency, duration	subjective	S	A		95.84	0.8182	90.28	0.5758
Plan of Management	Therapy details	subjective	S	A		83.34	0.4353	83.33	0.4368
Plan of Management	Active program - planned/ detailed	subjective		A		79.17	0.5604	62.50	0.1914
Plan of Management	Evidence-based	objective		A		95.84	0.73333	86.11	0.3182
Plan of Management	Complexity Completed	objective		A		87.50	0.6418	88.89	0.6883
Plan of Management	Dx & POM - Verified by clinician	objective			E	91.67	0.6364	83.33	0.4525
Goals & Outcomes	Appropriate Specific & measurable goals & outcomes present	objective		A		75.00	0.4217	63.89	0.0517
Goals & Outcomes	Repeat OM measures administered	subjective		A		66.67	0.3786	51.39	0.1286
Prognosis	Prognosis appropriately defined	subjective	S	A		91.67	0.7209	80.56	0.4540
Prognosis	Short term & long term prognosis, support by positive and negative prognostic factors	subjective	S	A		75.00	0.5826	58.33	0.3463
Consent / ROF	ROF complete	subjective	S	A		87.50	0.6539	80.56	0.5512
Consent / ROF	Risks – major/minor & benefits	subjective	S	A		91.67	0.7176	91.67	0.7512
Consent / ROF	Recording of patient Questions in ROF	objective	S			87.51	0.7714	97.22	0.9428
Consent / ROF	ROF signed by patient	objective	S			95.83	0.8571	94.44	0.8093
Consent / ROF	ROF signed by clinician	objective	S			95.83	0.8571	94.44	0.8093
Consent / ROF	ROF/IC current	objective	S	A		95.83	0.8571	94.44	0.8093
Consent / ROF	IC signed by patient:	objective	S			95.83	0.8571	91.67	0.7323
Consent / ROF	IC signed by clinician	objective	S			95.83	0.8571	94.44	0.8093
Consent / ROF	IC signed by intern	objective			E	95.83	0.8571	94.44	0.8093
Case Report / Physicians letter	Clear concise narrative	subjective			E	70.83	0.4167	63.89	0.1628
Case Report / Physicians letter	Professional format	subjective			E	75.00	0.3077	76.39	0.2842
Documentation	Collaborative care/correspondence (if applicable)	subjective		A		75.00	0.4667	70.83	0.2738
Documentation	Exchange of Medical Info form present & complete	objective			E	87.50	0.8059	34.72	−0.0349
Documentation	Dashboard (brown) boxes current & complete (Social Hx, Med.Hx, Ongoing Concerns, Reminder Section)	subjective	S	A		70.84	0.4894	66.67	0.3410
Documentation	Time to conditional case sign-off < 7 days	subjective			E	50.00	0.3043	30.56	−0.0164
Documentation	Case Signed off	objective			E	87.50	0.5714	73.61	0.2392
Documentation	SOAP notes: - Complete	subjective	S			87.50	−0.0588	77.78	−0.0159
Documentation	Clinician verification of SOAP	objective	S			95.83	0.0000	94.44	−0.0286
Documentation	Response to care documented in SOAP	subjective	S			79.16	0.5200	66.67	0.1787
Documentation	Timely uploading of documents	objective	S	A		62.50	0.2703	63.89	0.2034
Documentation	Compliance documented in file	subjective	S	A		66.66	0.4037	48.61	−0.0238
Re-evaluation	Completed as scheduled in POM	subjective	S	A		87.50	0.8059	29.17	−0.0588
Re-evaluation	Includes relevant details - Thorough & Complete	subjective	S	A		74.99	0.6308	29.17	−0.0217
Totals			*N* = 38	*N* = 37	*N* = 10	*N* = 61	N = 61	N = 61	N = 61
> 70% agreement per Standards: test-retest			32/38 (84%)	31/37 (84%)	9/10 (90%)	52 > 70%	k > 0.6 32/61	37 > 70%	k > 0.6 16/61
> 70% agreement per Standards: IRR			23/38 (60%)	23/37 (60%)	5/10 (50%)				

*ROF* report of findings. *SP* Standards of practice. *AS* Accreditation standard. *ES* Educational standard. *IRR* interrater reliability

Inter-rater element analysis revealed overall 70% or better agreement on 37 of 61 items (60%), and only 16/61(26%) items had K 0.6 or higher (range = − 0.21 to 1.00). With respect to the objective and subjective categories, 16/20 items (80%) and 21/41 items (51%) respectively attained 70% agreement or better (Table 3).

Although most Standards of Practice and Accreditation Standards items coincide, only 38/61 and 37/61 items respectively account for the total number of items in each set of standards, an additional 10 comprising individual institutional requirements. Element analysis according to each set of standards, revealed 32/38 (84%), 31/37 (84%) and 9/10 (90%) items achieving a minimum test-retest agreement level of 70% for Practice and Accreditation Standards and institutional requirements, respectively. In comparison, the corresponding values for inter-rater reliability were even lower at 23/38 (60%), 22/37 (60%) and 5/10 (50%) (Table 3).

### File scores

**Fig. 1:**
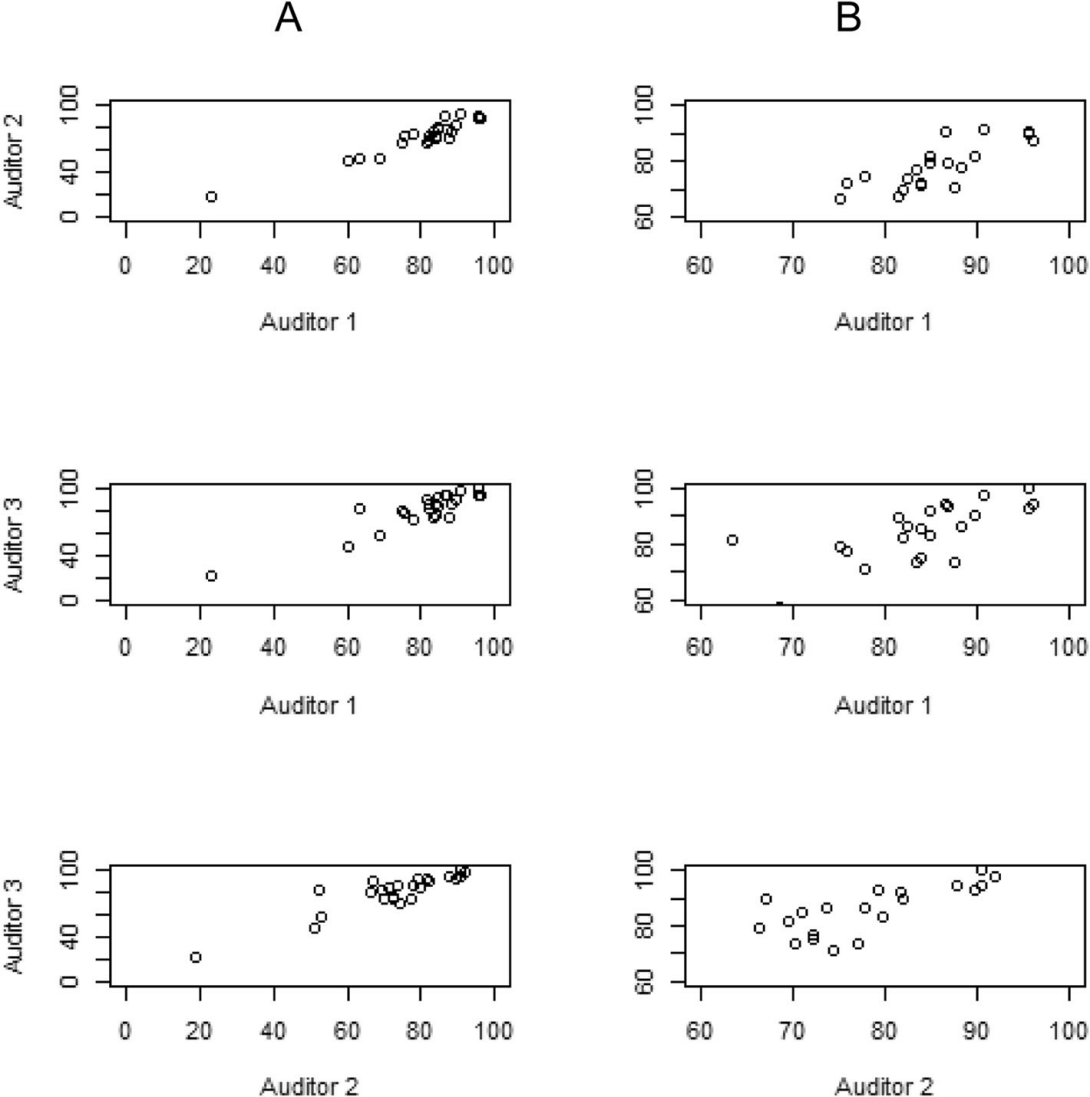
Scatterplots for inter-auditor reliability of Standards of Practice Scores, based on *n* = 24 files on the left (**A**) and *n* = 23 files on the right (**B**. one outlier removed)

For each of the 24 files audited, scores were generated pertaining to the domains of Standards of Practice, Accreditation Standards as well as overall file scores. Scatter plots of the scores revealed scores for one of 24 files to be an outlier and so scatter plots and ICCs were computed both including and excluding these scores in the analysis. For example, Fig. 1 represents the scatter plots for Standards of Practice scores, illustrating the influence of the outlier. Excluding it indicates the range of scores for the 23 included files are more representative of a typical audit outcome (Fig. 1, right panel). The ICC values as well as 95% CI for the 23 files indicate moderate correlation coefficients and fairly wide CIs (Table 4). The ICC values with the outlier record included were considerably higher due to increased file to file variability in the scores with this record included.

**Table 4:** Reliability of audit scores for sample, outlier removed (*N* = 23 files)

	Test-retest	Interrater
Standards of Practice Score	!CC(2,1) = 0.67	ICC (2,1) = 0.70
	95% CI (0.36, 0.84)	95% CI (0.36, 0.87)
Accreditation Score	ICC(2,1) = 0.76	ICC (2,1) = 0.56
	95% CI (0.51, 0.89)	95% CI (0.27, 0.77)
Overall Score	ICC(2,1) = 0.68	ICC (2,1) = 0.69
	95% CI (0.39, 0.85)	95% CI (0.45, 0.85)

### Audit times

One observation in the audit times stood out as an outlier at 161 min. For the remaining data, the mean time taken to audit a patient file was 20.0 (SD 9.3) minutes, with a range of 6 to 54 min. There were no significant differences between the auditors in time taken to audit files.

## Discussion

In this study we did not establish a priori criteria as threshold(s) of reliability [38]. However, for the purpose of analysis and discussion we have chosen the well-established value > 0.6 K to indicate better than substantial reliability [34] and 70% as a minimally acceptable level of PA. Higher PA values are of course desirable. However, interpretation of PA varies depending on the discipline under investigation. For example, in an investigation of chart abstraction in an urgent care setting the benchmark was set at 95% [5]. Regarding the instrument under scrutiny in this study, our results indicate substantial level of intra-rater reliability with 89% agreement and K of 0.75 across all 20 objective items, and 82% and K of 0.65 across all 41 subjective items for all three auditors (Table 1). However, determinations for inter-rater reliability were less encouraging, a moderate K value of 0.59 for all 20 objective items, and 0.44 across all 41 subjective items being obtained. The respective PA values were also correspondingly lower, at 82% for objective, and 70% for subjective items (Table 2).

Element analysis indicated overall 85% (52/61) of the items had intra-rater agreement of 70% or better across all three auditors. In comparison, inter-rater agreement was poor, only 60% (37/61) of items achieving or exceeding this arbitrary benchmark. K values both for intra- and inter-rater reliability were typically low, the lowest overall K being determined for inter-rater reliability; only 26% of the elements achieving 0.6 or higher, while some items had a negative K indicating no agreement at all (Table 3). It is difficult to interpret or explain the low K values reported for individual items, more so where PA is high (e.g., Clinician verification of SOAP notes: 94.4%, K – 0.03). This paradox of high PA and low K has been recognized and factors contributing to its occurrence have been identified and discussed in detail [39, 40]. It is suggested that K is influenced by factors that include the number of elements in the categories examined as well as the effect of prevalence. Our audit instrument contained a large number of items and most items were by definition representative of attributes whose presence in the record was a requirement, which students were expected to complete. The high prevalence of such items matches the scenario leading to K paradox identified in the literature [39–41] and indeed the result highlighted above, “Clinician verification of SOAP notes”, had 23/24 ratings of complete for both ratings and only 1/24 rating of not complete by one rater. These factors may have contributed to relatively high observed PAs, while decreasing K values. Direct comparison of inter-auditor reliability observed in the current study with reliability studies in the literature is not possible, as our search of the literature failed to identify reliability studies of audit instruments and processes assessing clinical records of entire patient files in the educational setting. Most studies have examined the reliability of audit instruments, attaining substantial-to-excellent inter-rater reliability, in assessing data abstraction of specific features of clinical notes [4, 6–11, 42] such as records related to asthma in a multicenter asthma care program [7], cardiovascular disease in primary care [4], or SOAP notes in an inpatient internal medicine setting [11]. In contrast, the instrument under scrutiny in this study is a checklist, comprised of elements in a structured curriculum rather than constructs aimed at assessing single domains or categories such as “history” or “diagnosis. Two clinical record audit studies in the area of chiropractic patient care have assessed quality of clinical documentation, one in an educational setting [20] and the other in the setting of professional practice across the US [22]. However, no details of the instrument used was provided in either case, although the latter study reported the instrument used had acceptable reliability indicators.

In the current study, overall inter-rater reliability is poor, particularly in the subjective category. The low reliability ratings of individual elements, both in terms of PA and K values are reflective of this outcome and may cast doubt on the validity of the instrument. It is not within the scope of this manuscript to scrutinize each and every element. However, in the interest of improving the audit instrument and thereby the audit process, it is important to highlight some aspects of the instrument, which may have contributed to the observed low PA and K values. It has been suggested that elements requiring clinical interpretation may diminish inter-rater reliability [5]. Indeed, scrutiny of the data in Table 3 indicates that some elements may require a response based on an auditor’s clinical judgment. For example, the elements “diagnosis was adequate”, Physical Examination “Procedures sufficient to exclude differential diagnoses” or the Plan of Management was “evidence based” (Table 3) would have all required some degree of clinical interpretation on the part of auditors and may have led to variability in responses. There are also several examples of low-to-very low inter-rater agreement due to ambiguity of elements. For example, the first 3 elements in the history section (Table 3, *Demographic Data Complete; Privacy Form present and complete; New Patient Form present and complete),* which are designated as being “subjective” could/should be altered to the “objective” yes/no designation provided that a clear explanation is given to the auditors as to what “complete” means in the context of the audit. Similarly, in the Documentation section, the element, inquiring if SOAP notes were “complete” (Table 3) may be confusing. Responses to such an open question would likely vary depending on how an auditor would interpret the word “complete” in the context of SOAP notes. Thus, rigorous training of auditors and precise explanations to guide auditor decisions are required in order to improve audit outcomes [4, 11]. Other elements might have created confusion for auditors with respect to their interpretation or location in the EHR. Plan of management and informed consent forms, which require multiple signatures are often initially done on paper. If such items are not scanned into the EHR in a timely manner, they would not be electronically extracted by the instrument and would not have been “seen” by the auditors unless they made the extra effort of searching for them in the original files in the EHR. Failure of auditors to navigate and locate required information in the EHR using a uniform approach might have contributed to poor inter-rater reliability [42].

Further Scrutiny of Table 3 indicates that most but not all Standards of Practice and Accreditation Standards items coincide. This is not surprising as they have legislative and academic mandates respectively in the development of future health care providers. It is interesting that some elements not covered by either set of standards, have been included as institutional requirements, including “range of motion” and “palpation”, both considered to be important in the context of teaching in a chiropractic curriculum. An estimate of the consistency with which elements representing Standards in each of the 23 files vis a vis intra or inter-rater reliability was obtained (Table 4). Relatively low ICC values for file sores in each of the domains of Standards of Practice or Accreditation Standards, as well as overall file scores suggest considerable heterogeneity in documentation of clinical notes and may explain the relatively low reliability ratings. This may negatively impact effectiveness of any feedback to clinicians. The effectiveness of audit and feedback has been reviewed and factors contributing to effective feedback have been suggested [2]. Clearly the reliability of the audit instrument used to provide feedback is one such factor.

On the basis of the results of this study and our analysis, it appears inter-rater reliability and generally the audit process will improve with specific attention to designation of elements to objective or subjective categories, and preparation of a guidebook to provide auditors with direction. It may be necessary to have consultative sessions with all steakholders including clinic management administrators, auditors, clinician reps as well as IT experts, to review and consider each and every element and make changes in the light of results presented in this study. We feel this approach would help minimize potential variability in interpretation of constructs comprising the checklist. The use of a guidebook will also assist in auditor training. Not only this is an important activity to enhance uniformity in interpretation, but also it is crucial in recruiting and training new auditors and ensuring continuity. Finally, efforts should be made to increase uniformity in documenting, uploading and storing data in a standardized fashion in order to minimize heterogeneity in patient files of different clinicians. We feel these suggested measures would help improve inter-rater reliability and ultimately contribute to the audit/feedback process.

### Strengths & Limitations

The choice of auditors for this study was very pragmatic. All three auditors were experienced clinicians and routinely did the audits. This ensured the authenticity of the process and eliminated the need to train new auditors. The less than satisfactory inter-rater reliability reflects inherent drawbacks of the audit instrument. Although as indicated in the methods section, the face and content validity of the instrument was established through the consultative phase of its development, no attempt was made to obtain a priori evidence of all features of validity [34] In this context, it is a strength that the instrument used is a specially developed checklist within the software, designed to assess the specified elements drawn directly from the EHR. As such it is readily modifiable and will potentially exhibit higher levels of reliability in an updated version, and will facilitate validating the instrument further.

The sample size of 24 files was chosen consistent with the clinics’ routine file audit procedure, and in retrospect may have been too small. However, considering element (item) level data were used to determine reliability, sample size was robust, 480 objective and 984 subjective elements were included in analysis.

The instrument used contained a large proportion of subjective items, which likely led auditors to apply their own clinical interpretation or use cognitive judgment in their responses. The fact that an accompanying guide was not provided specifically clarifying the intent of some elements that required interpretation may have contributed to the sub-optimal outcomes observed in the current study. The use of a detailed guide, whether in the context of regulatory, practice-based audits (3) or data abstractions in the clinical setting (4, 11), has been recommended.

## Conclusion

The aim of our study was to determine if the instrument used for patient file audits, and thereby the process of file audits at CMCC clinics, is adequate. The results indicate that overall, the audit instrument under study has substantial intra-rater reliability and poor-to-moderate level of inter-rater reliability. Several recommendations are made to improve inter-rater reliability of the audit instrument.

## Supplementary Information

**Additional file 1:** The Audit Instrument.

## Data Availability

Data used in this study is available from the corresponding author upon reasonable request.

## Abbreviations

CMCC: Canadian memorial chiropractic college
EHR: Electronic health record
IT: Information technology
PA: Percent agreement
K: Kappa
CI: Confidence interval
ICC: Intraclass coeficient
