# Supplementary material for: Intra- and inter-rater reliability of an electronic health record audit used in a chiropractic teaching clinic system: an observational study
Source: BMC Health Serv Res. 2021 Jul 28;21:750. doi: 10.1186/s12913-021-06745-1 (PMC8317378; doi:10.1186/s12913-021-06745-1)
Supplement: Supplementary file 1 — Additional file 1: The Audit Instrument. [file 12913_2021_6745_MOESM1_ESM.docx]

The Audit Instrument

|  | **Section** | **Response type** | **Standards of practice** | **Accreditation**  **standard** | **Educational standard** |
| --- | --- | --- | --- | --- | --- |
| **History** | Demographic Data Complete | subjective | S |  |  |
| History | Privacy Form present and complete | subjective |  |  | E |
| History | New Patient Form present and complete | subjective | S |  |  |
| History | Health Status Survey/Pain Diagram reviewed by clinician | objective | S |  |  |
| History | Outcome Measures appropriate | objective | S | A |  |
| History | Past Health History | subjective | S | A |  |
| History | Family Health History | subjective | S | A |  |
| History | Social History | subjective | S | A |  |
| History | Flags | subjective |  | A |  |
| History | Primary Complaint Complete | subjective | S | A |  |
| History | Secondary Complaint (if applicable) | subjective | S |  |  |
| History | Consent to Physical Examination obtained and documented | subjective | S | A |  |
| History | Differential Diagnoses rendered | subjective | S | A |  |
| History | Complexity Completed | objective |  | A |  |
| **Physical Examination** | Observation/posture | subjective | S |  |  |
| Physical Examination | Vitals | subjective | S | A |  |
| Physical Examination | Range of Motion | subjective |  |  | E |
| Physical Examination | Palpation | subjective |  |  | E |
| Physical Examination | Orthopaedic Procedures | subjective | S | A |  |
| Physical Examination | Correlates with history | subjective |  | A |  |
| Physical Examination | Neurologic Procedures | subjective | S | A |  |
| Physical Examination | Procedures sufficient to: Exclude differential diagnoses | subjective |  | A |  |
| Physical Examination | Procedures sufficient to: Render clinical diagnosis | subjective | S | A |  |
| Physical Examination | Complexity Completed | objective |  | A |  |
| **Diagnosis** | Diagnoses rendered | subjective | S | A |  |
| Diagnosis | Appropriate and supported by findings | subjective |  | A |  |
| Diagnosis | Complexity Completed | objective |  | A |  |
| **Plan of Management** | Further Evaluation | subjective | S |  |  |
| Plan of Management | Frequency, duration | subjective | S | A |  |
| Plan of Management | Therapy details | subjective | S | A |  |
| Plan of Management | Active program - planned/ detailed | subjective |  | A |  |
| Plan of Management | Evidence-based | objective |  | A |  |
| Plan of Management | Complexity Completed | objective |  | A |  |
| Plan of Management | Dx & POM - Verified by clinician | objective |  |  | E |
| **Goals & Outcomes** | Appropriate Specific & measurable goals & outcomes present | objective |  | A |  |
| Goals & Outcomes | Repeat OM measures administered | subjective |  | A |  |
| **Prognosis** | Prognosis appropriately defined | subjective | S | A |  |
| Prognosis | Short term & long term prognosis, support by positive and negative prognostic factors | subjective | S | A |  |
| **Consent / ROF** | ROF complete | subjective | S | A |  |
| Consent / ROF | Risks – major/minor & benefits | subjective | S | A |  |
| Consent / ROF | Recording of patient Questions in ROF | objective | S |  |  |
| Consent / ROF | ROF signed by patient | objective | S |  |  |
| Consent / ROF | ROF signed by clinician | objective | S |  |  |
| Consent / ROF | ROF/IC current | objective | S | A |  |
| Consent / ROF | IC signed by patient: | objective | S |  |  |
| Consent / ROF | IC signed by clinician | objective | S |  |  |
| Consent / ROF | IC signed by intern | objective |  |  | E |
| **Case Report / Physicians letter** | Clear concise narrative | subjective |  |  | E |
| Case Report / Physicians letter | Professional format | subjective |  |  | E |
| **Documentation** | Collaborative care/correspondence (if applicable) | subjective |  | A |  |
| Documentation | Exchange of Medical Info form present & complete | objective |  |  | E |
| Documentation | Dashboard (brown) boxes current & complete (Social Hx, Med.Hx, Ongoing Concerns, Reminder Section) | subjective | S | A |  |
| Documentation | Time to conditional case sign-off < 7 days | subjective |  |  | E |
| Documentation | Case Signed off | objective |  |  | E |
| Documentation | SOAP notes: - Complete | subjective | S |  |  |
| Documentation | Clinician verification of SOAP | objective | S |  |  |
| Documentation | Response to care documented in SOAP | subjective | S |  |  |
| Documentation | Timely uploading of documents | objective | S | A |  |
| Documentation | Compliance documented in file | subjective | S | A |  |
| **Re-evaluation** | Completed as scheduled in POM | subjective | S | A |  |
| Re-evaluation | Includes relevant details - Thorough & Complete | subjective | S | A |  |
